# DECREASING PAIN, ANXIETY, AND CONFUSION IN TERMINALLY ILL VETERANS

**DOI:** 10.1093/geroni/igac059.3054

**Published:** 2022-12-20

**Authors:** Denise Kresevic, Muralidhar Pallaki, Bridgette Wasil, Marilyn Swanson, Christopher Burant

**Affiliations:** VA Northeast Ohio Healthcare System, Sagamore Hills, Ohio, United States; VA Northeast Ohio Healthcare System, Cleveland, Ohio, United States; VA Northeast Ohio Healthcare System, Cleveland, Ohio, United States; VA Northeast Ohio Healthcare System, Cleveland, Ohio, United States; Case Western Reserve University, Parma, Ohio, United States

## Abstract

Terminally ill patients often receive medications for pain and anxiety that result in sedation that may inhibit communication with their loved ones. Balancing comfort while maintaining meaningful communications is a common dilemma for patients, staff, and families. Many patients report confusion and a decreased ability to communicate with families and health care providers, resulting in fear, anger, and frustration. Additionally, family members (25 to 33%) also feel frustrated and angry not being able to communicate with their loved ones needs and wishes. Delirium, a distressing syndrome characterized by disturbed consciousness, reduced ability to focus attention, altered cognition is experienced by 62–88% of terminally ill patients. Unfortunately, it is often underdiagnosed and undertreated. While not all delirium can be prevented it is estimated that 50% of terminal delirium may be ameliorated. This project was designed to proactively identify terminally ill veteran preferences for nonpharmacological interventions to reduce pain, anxiety, and prevent delirium. In the sample of Veterans from an inpatient VAMC Hospice (n=31) the mean age was 79.46 (sd=10.69); 60.7% were Black and 39.3% were white; 60.9% had cancer and 27.6% had heart failure. The most common patient delirium related behaviors were verbal agitation, physical agitations, physical aggression, anxiety, and confusion. Veterans identified the following comfort priorities: decreased noise (82.6%) and lighting (82.6%), warm blankets (78.3%), music (65.2%), emotional support (73.9%), increased family involvement (43.5%) and (20.8%) going outdoors. Preventative individualized nonpharmacological interventions decreased disruptive delirium related behaviors over 95% of the time and should be incorporated into routine hospice care.

